# Editorial: The cross-talk between gut microbiota and endogenous metabolites in endocrine diseases, volume II

**DOI:** 10.3389/fendo.2023.1320646

**Published:** 2023-11-08

**Authors:** Van B. Lu, Guilherme Zweig Rocha

**Affiliations:** ^1^ Department of Physiology and Pharmacology, University of Western Ontario, London, ON, Canada; ^2^ Department of Internal Medicine, School of Medical Sciences, University of Campinas, Campinas, Brazil

**Keywords:** microbiota, endocrine, diabetes mellitus, chronic liver disease, enteroendocrine cell

The gut microbiota is a community of micro-organisms that inhabit a host’s gastrointestinal tract. Over a trillion microbes contribute to shaping this environment through the production of metabolites that can impact host physiology and function. Environmental factors as well as host attributes such as sex and age are important determinants of gut microbial composition and the metabolites they produce. There are a growing number of microbially-produced metabolites that have been associated with positive host health; however, the mechanisms and signaling pathways these metabolites activate have yet to be fully elucidated. Understanding these mechanisms will allow the field to better understand this intricate symbiotic relationship between gut microbiota and humans.

Dysbiosis, or the imbalance of gut microbial communities, can disrupt host health and can even lead to several disease states, including endocrine disorders. The endocrine system involves the release of hormones from glands that enter blood circulation to exert a wide range of effects on growth, development, metabolism, sexual function and mood. The indispensable nature of a functioning endocrine system is exemplified by deficiencies or excess production of hormones in circulation that lead to endocrine disorders. Circulating levels of bacterial metabolites can induce or influence the occurrence of endocrine disorders. The emergence of pharmacotherapies aimed at reshaping host-microbiota interactions has added a new dimension for managing endocrine diseases and highlight the importance of this reciprocal relationship.

The present Research Topic builds upon the first edition of the Research Topic – *The Cross-Talk Between Gut Microbiota and Endogenous Metabolites in Endocrine Diseases*, with an update on this rapidly growing field. The goal of this topic is to identify the origin of endogenous metabolites generated by gut flora and its effects on endocrine physiology. This includes uncovering novel associations between endogenous metabolites produced by gut microbiota and host endocrine system and determining the signaling pathways recruited by these metabolites.

Diabetes mellitus (DM) is the most common endocrine disease, afflicting over 400 million people worldwide across socio-economic boundaries. DM is also an endocrine disease linked with dysbiosis of the gut microbiota (1). Several studies in this Research Topic examine novel aspects of this disease and gut microbes. The observational study from Guo et al., examines oral microbiota and its relationship with intestinal microbiota in patients with type 2 DM. Dysbiosis of oral flora and transmission to the intestine provide novel avenues for diagnosing and treating type 2 DM. A systematic review from Hong et al., investigates the involvement of gut microbiota and incidence of diabetic microvascular complications (DC). DC complications include diabetic retinopathy and diabetic peripheral neuropathy, which contribute to the high mortality and morbidity rate in DM patients. Their study found depletion of short-chain fatty acid-producing bacterial richness was associated with DC, and these results can inform future studies into the progression of DM to more severe disease states. Finally, the study by Ma et al. characterizes changes in the gut microbiota in a rat model of DM following treatment with Yu-Ye Tang (YYT). YYT is a widely used, traditional Chinese medicinal product used to treat diabetes and gastritis, but its specific mechanism of action is unclear. This study combines *in vivo* measurements of host endocrine function and uses methods to detail changes in the microbial community and metabolites during disease and treatment with YYT. This study also provides a useful framework to investigate the effects of herbal formulations on modulating gut microbiota and host metabolism in future studies.

Endocrine disorders also have a huge impact on liver function. Although many studies, including ones included in this Research Topic, have drawn associations between dysbiosis and DM, the link between gut microbiota and chronic liver disease is more controversial. The study by Zhang et al. adds clarity to this issue by using a novel approach to combine host genetic variants to explore the causal association between gut microbial populations and metabolites with chronic liver diseases.

Tackling the underlying mechanism of action of bacterial metabolites on host health, the final two submissions examine mechanisms involving the hormone releasing cells of the gut, the enteroendocrine cells. Larraufie et al. examines the gasotransmitter hydrogen sulfide, which is generated in the gut by sulfate-reducing bacteria, and its effects on enteroendocrine cell metabolism and function. Finally, the review by Masse and Lu covers the main bacterial metabolites produced in the gut and the known signaling pathways recruited in enteroendocrine cells. The utility of targeting these mechanisms as a treatment for metabolic diseases such as diabetes and obesity are also covered in this comprehensive review.

This Research Topic spans the breadth of current research at the intersection of endocrinology and gut microbiology. These studies advance our understanding of the impact changes in gut microbial populations have on host endocrine function and provides insight on potential therapeutic strategies targeting microbial populations to improve human health.

## Author contributions

VL: Writing – original draft, Writing – review & editing. GR: Writing – review & editing.
